# Gynecologists Need to Be Vigilant—Two Case Reports of Intravascular Leiomyomatosis and Literature Review

**DOI:** 10.3389/fonc.2022.840096

**Published:** 2022-02-07

**Authors:** Xiang Li, Ning-Ye Ma, Yao Zhang, Yi-Sheng Jiao

**Affiliations:** Department of Obstetrics and Gynecology, Shengjing Hospital of China Medical University, Shenyang, China

**Keywords:** intravascular leiomyomatosis (IVL), misdiagnosis, recurrence, treatment, operation

## Abstract

**Background:**

Intravascular leiomyomatosis is a rare benign lesion with malignant potential. The cases are sporadic. Most patients have no clinical symptoms, and the preoperative diagnostic rate is low. Case 1 was misdiagnosed, passively managed during operation, recurred quickly, and underwent a secondary operation. We learned lessons from case 1 and treated the case 2 patient differently. The case 2 patient had a good prognosis. We hope the report will be helpful to other gynecologists.

**Case Summary:**

Case 1: a 49-year-old woman complained of dysmenorrhea. Traditional ultrasound showed adenomyosis and a solid mass 6 * 3 cm in the right appendix. After routine examination, the patient underwent transabdominal total hysterectomy + bilateral salpingectomy + IVL tumor resection, with both ovaries kept. No medication was used after operation. Routine ultrasound was performed every 3 months. The disease recurred, and the patient underwent a secondary surgery 9 months after the first time. So far, 25 months after the secondary surgery, there is no sign of recurrence. Case 2: a 41-year-old woman underwent a routine body examination, where a left adnexal mass 7 cm was found. The patient underwent contrast-enhanced ultrasonography and was diagnosed and prepared well preoperatively. The patient underwent transabdominal total hysterectomy + bilateral salpingectomy + IVL tumor resection. GnRH-a drugs were used after operation for 3 cycle. Now, there is no sign of recurrence after operation for 23 months.

**Conclusion:**

The incidence rate of IVL is low, and there are no typical clinical symptoms. It is easy to be ignored by gynecologists. Contrast-enhanced ultrasound is helpful to diagnose preoperatively and reduce misdiagnosis. Good preparation, full exploration of the pelvic and abdominal vessels, removal of lesions completely as much as possible, and anti-estrogen therapy after operation can reduce the recurrence of disease.

## Introduction

Intravascular leiomyomatosis (IVL) is a special type of uterine myoma. The preoperative diagnosis rate is low because of no specific symptom, which leads to misdiagnosis. Inadequate pelvic exploration and missed lesions will increase the recurrence rate. This paper summarizes the experience and lessons from one misdiagnosed case; the second case is fully prepared and the treatment effect is good. We review the relevant literature, hoping to provide help to other clinical colleagues.

### Case Introduction

Medical history of the two cases displayed in **Table 1**.

**Table 1:** Chief history of two cases.

	Case 1	Case 2
Age	49	41
Chief complaint	Increased menstrual volume and dysmenorrhea	Normal body examination found pelvic mass
Past history	Gravida 2, para 1 (cesarean section), abortion 1 No other medical history, no smoking, no drinking history No myoma/adenomyosis history	Gravida 2, para 1 (delivery), abortion 1 No other medical history, no smoking, no drinking history No myoma/adenomyosis history
Laboratory examination	Blood HCG(-) HB 69 g/l CA125: normal ECG (electrocardiogram): normal UCG (echocardiography): normal	Blood HCG(-) CA125: normal ECG (electrocardiogram): normal UCG (echocardiography): normal
Imagine examination	Routine transvaginal ultrasound: uterus 10 * 7 cm, the thickness of anterior wall 2.2 cm, posterior wall was about 3.4 cm. a solid tumor about 6.4 * 3.2 * 1.8 cm in the right accessory area, with unclear boundary and tortuous course. CDFI can detect blood flow signal	① Routine transvaginal ultrasound: 7 cm tumor at the left rear of the cervix, CDFI detected rich blood flow signals, recommended contrast-enhanced ultrasonography ② Contrast-enhanced ultrasonography: after intravenous injection of contrast agent, the intravascular enhancement of the tumor in the left rear of the uterus began 13 s later. After 17 s, the tumor parenchyma and the myometrium of the uterus increased synchronously, and the echo intensity was similar, suggesting that the tumor has a close relationship with the uterus and parauterine veins, IVL (**Figure 1**)?
Diagnosis	Adenomyosis, pelvic solid mass (subserosal myoma? Ovarian fibroma)?	Pelvic mass (IVL?)
Treatment	① Routine preoperative preparation ② Transabdominal total hysterectomy + bilateral salpingectomy + IVL tumor resection	① Adequate blood preparation ② Transabdominal total hysterectomy + bilateral salpingectomy + IVL tumor resection
Operation process	① The uterus was enlarged, regular shape, adenomyosis like. ② The bilateral ovaries were regular ③ There were no other masses. After a repeated exploration nearly an hour, a strip solid mass about 6 * 3 * 1 cm were detected at the right pelvic floor, along the blood vessel, close to the pelvic wall, which we almost missed. The boundary was still clear. We removed the mass, and the quick pathology was benign. After intraoperative explanation, the patient’s families asked to preserve both ovaries.	① Multiple uterine myoma ② Multiple tubal arteriovenous myomas in the left broad ligament, normal appearance of both ovaries ③ After a careful exploration of pelvic vessels, we found multiple striped hard nodules in the left ovarian vein, running in the pelvic infundibulum ligament; a total length reached about 8 cm. All lesions were completely removed (**Figure 2**).
Special part for IVL operation	① First operation, the IVL was located in the uterine artery near the beginning of the anterior branch of the internal iliac artery. Because the patient underwent hysterectomy, we ligated the outer end of the IVL, 1 cm away from the beginning of the artery, and removed the uterine artery and its internal IVL ② Secondary operation, the IVL recurred at the artery ligation end. We ligated and removed the IVL at the beginning of the uterine artery ③ Second operation, due to pelvic adhesion, the right ureteral mucosa was thin after separation. We placed right ureteral stent at the same time (removed the stent 3 months after operation in clinic)	① The patient underwent total hysterectomy and bilateral salpingectomy; the IVLs in the fallopian tube arteriovenous vessels in the broad ligament were directly removed ② IVL in the left ovarian vein, we freed the vein. The assistant pressed the proximal end of the blood vessel. The operator took a small incision, completely extracted 8 cm IVL focus, and sutured the blood vessel wall
Treatment after operation	Routine antibiotic use	Routine antibiotic use + medication of GnRH-a for 3 cycles (GnRH-a: leuprorelin acetate microspheres sustained release for injection 3.75 mg per 28 days for 3 cycles)
Follow-up	① Recurred and a second operation was undertaken (oophorectomy + IVL tumor resection) 9 months after first time ② So far, 19 months after the second operation, no sign of recurrence	So far, 22 months after the operation, there was no sign of recurrence.

**Figure 1:**
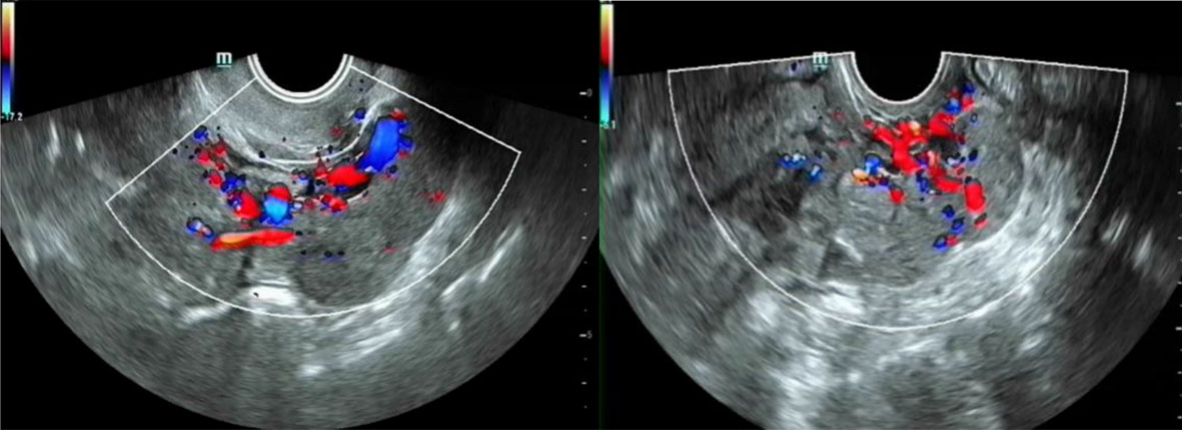
Contrast-enhanced ultrasonography imagine of case 2.

**Figure 2:**
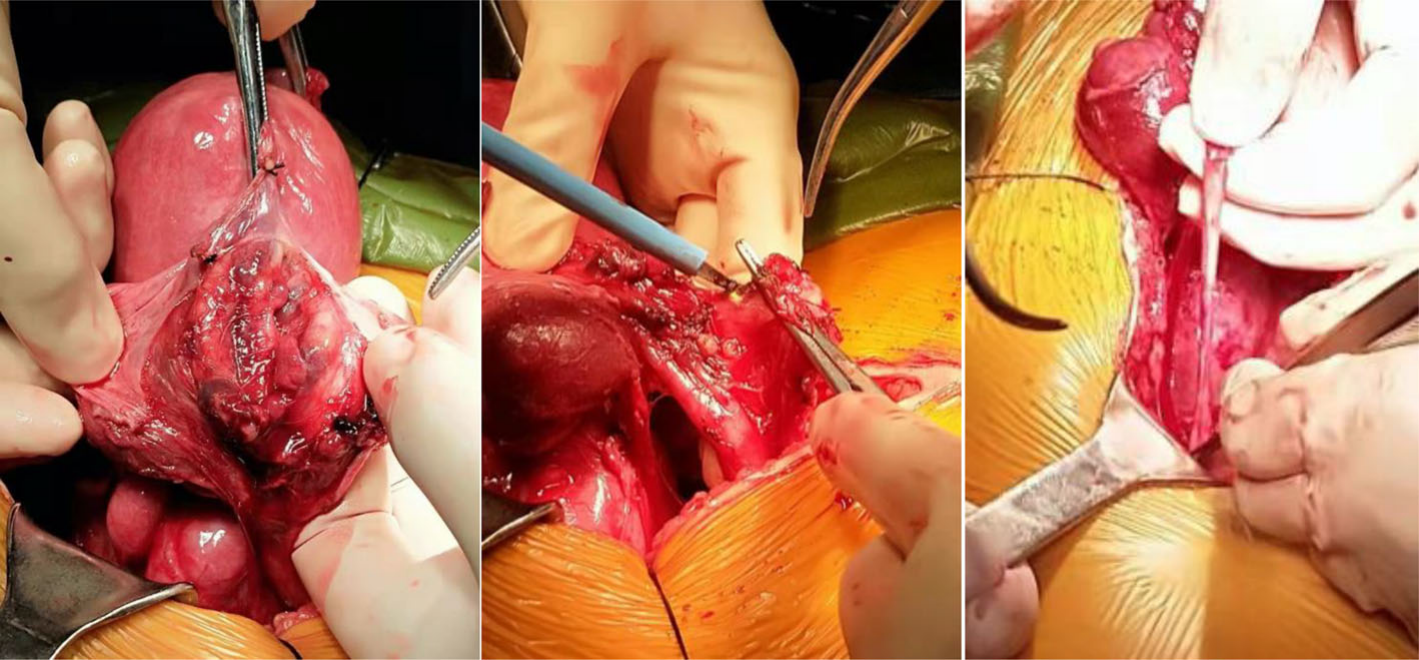
Intraoperative findings of case 2.

## Related Literature Learning

IVL mainly consists of benign smooth muscle tissue spreading and growing along the venous lumen. It is a rare special type of uterine myoma in gynecology. The onset age was 40–60 years, and 60% of the patients had a history of uterine myoma. The two patients we met had no previous history of hysteromyoma, but multiple hysteromyoma or adenomyosis was found in both cases during our operations. IVL invades not only the pelvic vein, inferior vena cava, renal vein, and pulmonary artery but also distant metastasis to the heart, lung, brain, and lymph nodes (1–3). The cases have been sporadic and mainly female. It is found that up to 10% of the cases invade the heart (4). Once the heart is involved, there may be symptoms such as palpitation, dyspnea, syncope, and edema of both lower limbs, and may even be life-threatening. At present, the cause of its pathogenesis is not completely clear. Most scholars believe that it is related to hormone level, local small injury, and venous congestion (5).

IVL has no obvious clinical symptoms and signs, and the preoperative diagnosis rate is very low. It is often misdiagnosed as uterine myoma, adenomyosis, pelvic malignancy, and so on. Our first case was misdiagnosed as uterine subserosal myoma or ovarian solid mass before operation. We were passive in finding the IVL lesion during operation, almost missing it. We spent nearly an hour to search the mass and only gave the patient routine blood preparation before operation. IVL is an estrogen-dependent benign disease and is recurrent. According to the intraoperative findings and quick frozen pathology, for older patients, bilateral oophorectomy can be considered to reduce recurrence. In our case 1, because the possibility of IVL was not fully considered before operation, we felt passive during the operation and almost missed the focus. The families were not fully prepared for oophorectomy during operation. Bilateral ovaries were preserved, and there was no medication after operation; the disease recurred half a year after operation. 9 months after the first operation, the patient took a secondary operation and experienced physical, psychological, and economic trauma again. During the second operation, because of pelvic tissue inflammatory adhesion, the operation was very difficult, and the risk of injury of adjacent organs such as the ureter is also greatly increased.

The advantages and disadvantages of common inspection methods are shown in **Table 2**. With the development of medicine, on the basis of ultrasound, the use of contrast agents has more and more advantages in the diagnosis of vascular-related diseases, especially in IVL. In this paper, the second patient was diagnosed clearly depending on the contrast-enhanced ultrasonography before operation and was fully prepared before operation; exploration during operation was comprehensive and supplementary medication after operation was timely. The prognosis was good.

**Table 2:** The traditional imaging detection of IVL.

Method	Describe	Advantage		Shortcoming
**Routine ultrasound**	The pelvic parauterine tissue has a mixed echo, unclear boundary, and rich blood supply, and internal blood vessels are in strip or dendritic shape, which should be differentiated from subserosal or broad ligament myoma and ovarian tumor	① Cheap and simple ② No radiation		It is easy to be misdiagnosed and should be differentiated from subserosal or broad ligament myoma and ovarian solid tumor
**CT**	The mixed density mass with an unclear boundary can be seen with a tortuous vascular shadow or vascular filling defect after enhancement		Continuous images are helpful for clinical doctors to read films	① CT has radiation (within the safe dose) ② Risk of contrast medium allergy
**MRI**	Irregular tortuous, shuttle, and solid mass in the myometrium or parauterine with multiple tortuous vessels in or near the tumor. DWI was high or slightly high, but the ADC value did not decrease significantly	① No radiation, multidirectional, clear anatomical relationship; soft tissue lesions are better than CT ② Continuous images are helpful for clinical doctors to read films		① Long time, confined space ② No pacemaker or contraceptive ring in the body ③ Risk of contrast medium allergy
**PET-CT**	The vascular lumen is tortuous and thickened, multiple uneven low-density shadows can be seen in or around the lumen, and FDG metabolic imaging can be seen locally or there is no abnormal radioactive concentration.	① Sensitivity, high specificity ② Systemic examination better for multiple and scattered lesions		Expensive

In general, the uterus of IVL can be enlarged, most of which are complicated with uterine myoma or adenomyosis. Tumors can be seen in the uterine wall, parauterine ligament, and adnexal vein, most of which are cord like, *Flammulina velutipes* like, and bead like, which can twitch. The section is gray, pink white, and soft or hard. Microscopically, the tumor cells were spindle shaped, braided, or vortex shaped, and the surface was covered with a single layer of flat vascular endothelial cells. There was no or occasional mitotic image. Previous reports suggested specific immunohistochemical markers, including tumor cells vimentin (+), desmin (+), and SMA (+), and endothelial cells of venous vessels CD34 (+), especially ER and PR (+).

IVL should be differentiated from uterine fibroids and ovarian cysts, as well as the following diseases under a pathological microscope (**Table 3**).

**Table 3:** Pathological differential diagnosis.

Disease name	
**Peritoneal disseminated leiomyoma**	It is composed of benign smooth muscle, but most of it is distributed in the peritoneum or serosa
**Endometrial stromal sarcoma** **(ESS)**	Cord-like masses can be seen in muscular vessels, usually without lobulation. Under the microscope, the cells were arranged disorderly and showed a basket-like structure under the electron microscope. Immunohistochemistry showed SMA (+), VIM (+), DES (+), CK (-)
**Uterine leiomyosarcoma with vascular invasion**	The sarcoma shows obvious heterotypic cells with coagulative necrosis and invasion of the surrounding muscle layer
**Uterine myxoid leiomyosarcoma**	It is generally gelatinous and brittle, with a fuzzy boundary. Under the microscope, the cells are significantly heterogeneous, secrete a large amount of mucus, and have intracellular mucus, and the boundary is often infiltrated

IVL is mainly treated by surgery. Because the tumor is diffuse and the recurrence rate is high, complete resection of the tumor is the key of surgery. Li et al. (6) sorted and analyzed the clinical data, pathological characteristics, and treatment methods of 194 IVL patients whose lesions extended to the heart publicly reported abroad. It was found that incomplete resection of IVL lesions would increase the recurrence rate and postoperative mortality. Wang et al. (7) also reported that the recurrence rate of complete resection was 21.4%, and that of incomplete resection was 50%. The difference was statistically significant.

Kokawa et al. (8, 9) found that ER and PR were positively expressed in IVL, and the estrogen receptor in IVL tissue was 10 times higher than that in normal tissue, and the progesterone receptor was 50 times higher. At the same time, the level of estradiol in serum was significantly increased (8). Therefore, in addition to surgical resection, some hormone drugs such as GnRH-a and aromatase inhibitor anti-estrogen therapy have also been introduced into IVL treatment. There is a debate about the therapeutic effect of hormone drugs in some literature. Lin et al. (6) reported that the use of anti-estrogen therapy after operation cannot prevent disease recurrence, but Bondner (10) and Nishizawa (11) reported that the preoperative application of anti-estrogen drug GnRH-a can reduce the IVL tumor volume by up to 50% and improve the surgical resection rate. Some scholars also reported that GnRH-a can be used to treat residual IVL and reduce the risk of recurrence (12, 13).

For IVL patients, preserving of the uterus and ovary, unclean lesion resection, application of estrogen drugs, lesion infiltration into parauterine vessels, or distant invasion can increase the recurrence rate. The treatment of IVL should focus on surgery and remove the lesions as much as possible. The preferred surgical method is total hysterectomy with bilateral salpingo-oophorectomy + extrauterine lesion resection. For young patients, to preserve reproductive function, the IVL tumors should be removed completely as much as possible, and GnRH-a drugs should be supplemented for follow-up treatment to reduce the risk of recurrence.

In conclusion, although IVL is sporadic, there are still many cases reported in recent years. Because of its sporadicity, it is difficult to arouse the vigilance of gynecologists, and routine gynecological preoperative ultrasound is difficult to make a clear diagnosis from, which often leads to inadequate preoperative preparation, wrong selection of operation methods, passive treatment during operation, and lesions being easily missed. Our cases suggest that clinical gynecologists should be vigilant and not only rely on routine ultrasound to assist in diagnosis and treatment. If the veins in broad ligament, parauterine tissue, or adnexal area are found to be cord-like or *Flammulina velutipes*-like changes during operation, the disease should be considered, the blood vessels in the operation field should be fully explored, and the lesions should be completely and fully removed as much as possible. GnRH-a drugs or other anti-estrogen drugs can be used after operation to reduce the risk of recurrence. Regular reexamination and close follow-up are needed after operation.

## Author Contributions

XL is the first author. Y-SJ is the corresponding author. XL drafted the manuscript. N-YM collected the patients’ clinical data. YZ contributed to analyzing and interpreting the imagine findings. Y-SJ gave the patients’ operation and reviewed the literature. All authors contributed to the article and approved the submitted version.

## Conflict of Interest

The authors declare that the research was conducted in the absence of any commercial or financial relationships that could be construed as a potential conflict of interest.

## Publisher’s Note

All claims expressed in this article are solely those of the authors and do not necessarily represent those of their affiliated organizations, or those of the publisher, the editors and the reviewers. Any product that may be evaluated in this article, or claim that may be made by its manufacturer, is not guaranteed or endorsed by the publisher.
